# Mitochondrial translocation of EGFR regulates mitochondria dynamics and promotes metastasis in NSCLC

**DOI:** 10.18632/oncotarget.5736

**Published:** 2015-10-19

**Authors:** Ting-Fang Che, Ching-Wen Lin, Yi-Ying Wu, Yu-Ju Chen, Chia-Li Han, Yih-leong Chang, Chen-Tu Wu, Tzu-Hung Hsiao, Tse-Ming Hong, Pan-Chyr Yang

**Affiliations:** ^1^ Institute of Molecular Medicine, College of Medicine, National Taiwan University, Taipei 100, Taiwan; ^2^ Institute of Biomedical Science, Academia Sinica, Taipei 115, Taiwan; ^3^ NTU Center for Genomic Medicine, College of Medicine, National Taiwan University, Taipei 100, Taiwan; ^4^ Department of Internal Medicine, College of Medicine, National Taiwan University, Taipei 100, Taiwan; ^5^ Institute of Clinical Medicine, College of Medicine, National Cheng-Kung University, Tainan 701, Taiwan; ^6^ Institute of Chemistry, Academia Sinica 115, Taipei, Taiwan; ^7^ Master Program for Clinical Pharmacogenomics and Pharmacoproteomics, School of Pharmacy, Taipei Medical University 110, Taipei, Taiwan; ^8^ Department of Pathology and Graduate Institute of Pathology, College of Medicine, National Taiwan University, Taipei 100, Taiwan; ^9^ Department of Medical Research, Taichung Veterans General Hospital, Taichung 407, Taiwan

**Keywords:** cancer metastasis, EGFR, mitochondria dynamics

## Abstract

Dysfunction of the mitochondria is well-known for being associated with cancer progression. In the present study, we analyzed the mitochondria proteomics of lung cancer cell lines with different invasion abilities and found that EGFR is highly expressed in the mitochondria of highly invasive non-small-cell lung cancer (NSCLC) cells. EGF induces the mitochondrial translocation of EGFR; further, it leads to mitochondrial fission and redistribution in the lamellipodia, upregulates cellular ATP production, and enhances motility *in vitro* and *in vivo*. Moreover, EGFR can regulate mitochondrial dynamics by interacting with Mfn1 and disturbing Mfn1 polymerization. Overexpression of Mfn1 reverses the phenotypes resulting from EGFR mitochondrial translocation. We show that the mitochondrial EGFR expressions are higher in paired samples of the metastatic lymph node as compared with primary lung tumor and are inversely correlated with the overall survival in NSCLC patients. Therefore, our results demonstrate that besides the canonical role of EGFR as a receptor tyrosine, the mitochondrial translocation of EGFR may enhance cancer invasion and metastasis through regulating mitochondria dynamics.

## INTRODUCTION

Various vital cellular functions are executed in the double-membrane organelles, mitochondria, including energy production, redox status, generation of reactive oxygen species, control of cytosolic calcium levels, and initiation of apoptosis [1]. Mitochondrial dysfunction has been shown to be associated with many human disorders, including metabolic diseases, aging, nervous system diseases [2], cardiac disorder [3], and cancer progression [1]. Mitochondria are dynamic organelles that consistently fuse with each other to form a tubular shape and divide into smaller fragments [4]. Mitochondria dynamics are pivotal to functions and quality control of mitochondria [5] and is central to cellular outcomes linked to cell death, development, aging and diseases [6, 7]. Evidences show that the utility of different energy source can modulate mitochondrial structure [8, 9], and alteration of mitochondria morphology by Huntingtin proteins is correlated to ATP production in the neuronal cells [10, 11]. All these reports suggest that energy production by the mitochondria is correlated to mitochondrial dynamics. In addition, mitochondrial morphology and distribution in cancer cells is also related to cancer cell motility and invasiveness [6, 12, 13].

Epidermal growth factor receptor (EGFR) is a receptor tyrosine kinase, which is expressed on the cell surface to trigger downstream kinase signaling pathway via a ligand binding-mediated phosphorylation, and its auto-activation is correlated with tumor progression [14]. Upon EGF binding, some EGF-EGFR complexes are internalized through endocytosis and enter the cytosol to fuse with endosomes for degradation [15], and some complexes are recycled back to the cell surface [16, 17]. Although EGFR lacks specific organelle-targeting sequence, evidences showed that the translocated EGFR has been identified in other subcellular location. The nuclear EGFR translocation is triggered by EGF stimulation, which induce EGFR of plasma membrane to traffic to nucleus [15, 18, 19]. Recently, mitochondrial EGFR and the EGFR variant, EGFRviii, are reported to be constitutively active and present in the mitochondria of glioblastoma cells [20]. Compared to the well-studied nuclear EGFR, how EGFR enters the mitochondria and the exact functions of mitochondrial EGFR remain unclear.

In this study, we analyzed mitochondria protein composition of NSCLC cells with different invasive abilities, and found that EGFR is one of the candidates which may influence cancer invasion. Ectopic expression of mitochondria-targeting-EGFR was used for investigation of the effects on mitochondria and the following phenotypes *in vitro* and *in vivo*, and simultaneously we also studied the effects of endogenous EGFR on those phenomena by EGF stimulation. The underlying mechanisms of regulation of mitochondria dynamics by EGFR were revealed to be correlated with Mfn1. The results above provide new insights of EGFR in mitochondria dynamics and NSCLC metastasis.

## RESULTS

### EGFR expression in the mitochondria of NSCLC cells

**Figure 1:**
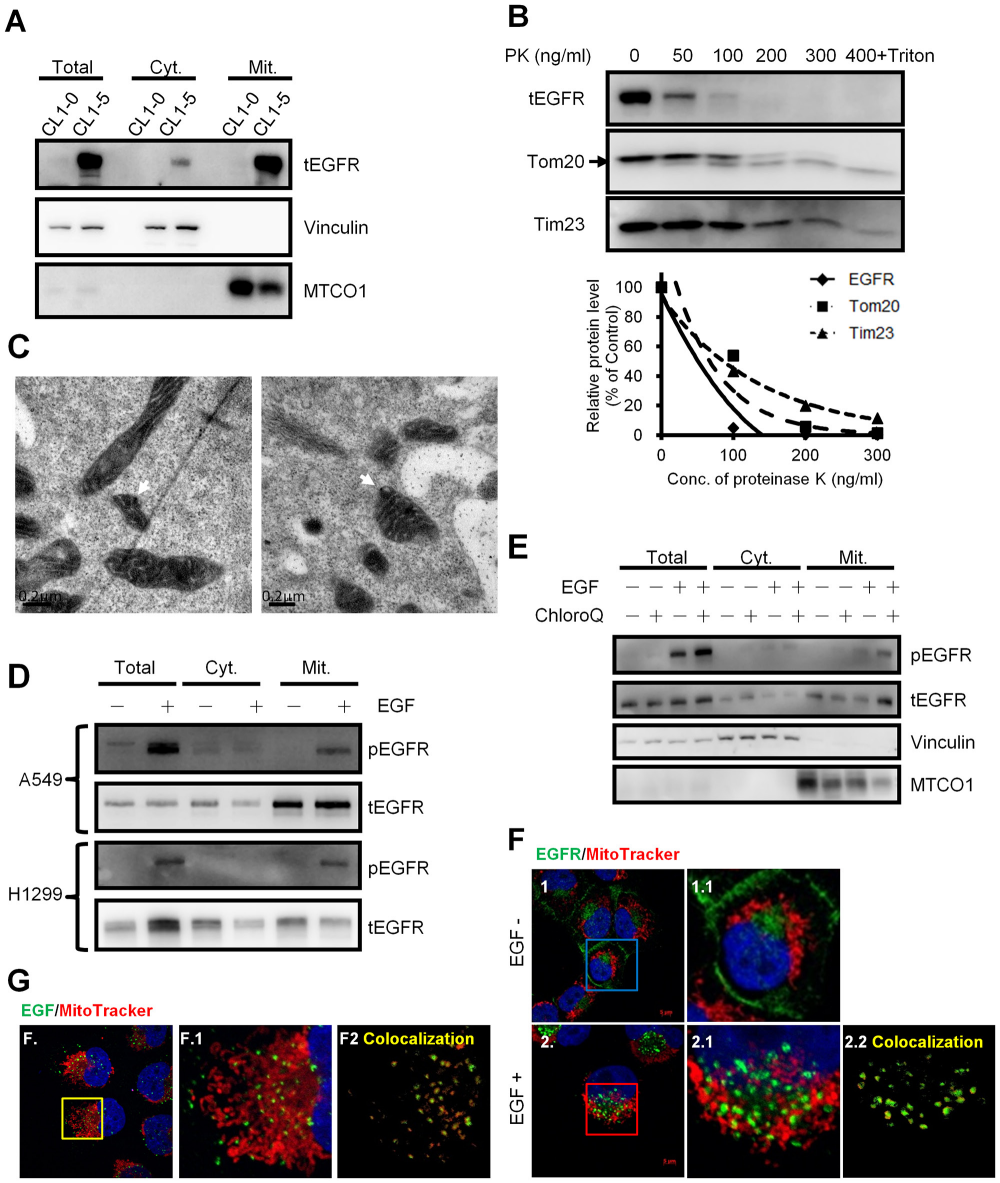
EGFR exists in the mitochondria of NSCLC cells and translocates through endocytosis **A.** Western blotting of the subcellular fractions of CL1–0 and CL1–5 cells. Vinculin and MTCO1 serves as a membranous, cytosolic marker and a mitochondrial marker, respectively. Cyt. and Mit. represent the cytosolic fraction without membranous organelles and the mitochondrial fraction, respectively. **B.** Proteinase K digestion assay for mitochondria fractions of H1299 cells. The intact mitochondria of H1299 was extracted and then incubated with the indicated concentration of PK at 25°C for 30 min. The fraction with PK and 0.1% of Triton X-100 serves as the negative control. After incubation, the mixture was subjected to immunoblotting. Tom20 and Tim23 serve as the markers for the outer and inner membrane of the mitochondria, respectively. The upper right panel showed the relative protein level digested by PK. **C.** H1299 cells were starved for 24 h, treated with40 ng/ml for 10 min., and then processed for immunoelectron microscopy analysis. White arrows indicate positive EGFR staining in the mitochondria. Scale bar: 0.2 μm. **D.** After serum starvation for 24 h, A549 and H1299 cells were treated with 40 ng/ml of EGF at 37°C for 30 min, and then subjected to the mitochondrial fractionation and immunoblotting. **E.** Starved H1299 cells were pretreated with 0.5 mM of chloroquine for 3 h at 37°C, and then treated with 40 ng/ml of EGF for 15 min. Then the treated cells were subjected into the mitochondrial fractionation and immunoblotting. **F.** H1299 cells were seeded on the coverslips and serum starved for 24 h. Cells were treated with 40 ng/ml of EGF and 200 nM of MitoTracker for 10 min. Anti-EGFR antibody was used for the immunofluorescence staining. The panels 1.1 and 2.1 are the magnified images of the square in the left panels. The panel 2.2 shows the signals of the colocalization of MitoTracker and EGFR analyzed by ZEN2009 software. Scale bar: 5 μm. **G.** H1299 cells were serum starved for 24 h and then treated with 200 ng/ml of EGF-Alexa Fluor 488 and 200 nM of MitoTracker for 10 min. The panel F.1 is the magnified image of the square in the left panels. The panel F.2 shows the signals of the colocalization of MitoTracker and EGF-Alexa Fluor 488 analyzed by ZEN2009 software. Scale bar: 5 μm.

To investigate the mitochondrial proteome in lung cancer cells with different invasive abilities, the mitochondrial protein from lung cancer cells CL1–0 and CL1–5, two of the cell lines derived from the same origins with different invasive abilities [21], were analyzed by iTRAQ as the flowchart shown in Supplementary Figure S1A. We found that EGFR expression was higher in the mitochondrial protein of the highly invasive CL1–5 comparing to the low invasive CL1–0 cells (Supplementary Table S1). The results were further confirmed by the immunoblot (Figure 1A). Vinculin [22], a membrane-cytoskeletal protein in the focal adhesion plaques, and MTCO1, a mitochondrial protein, were served as the membranous and cytosolic, and mitochondrial markers, respectively. The EGFR translocation in mitochondria could also be observed in other lung cancer cells regardless of their EGFR genotypes (Supplementary Figure S1B). Next, we examined the localization of EGFR in the mitochondria by proteinase K (PK) digestion. From Figure 1B, without destroying the lipid bilayer of the mitochondrial double membrane, the pattern of EGFR digested by PK was similar to that of Tom20 (the marker of mitochondrial outer membranes) but not Tim23 (the marker of mitochondrial inner membrane), indicating that EGFR is located on the outer membrane of the mitochondria. Furthermore, the electron microscopic images of H1299 cells in Figure 1C also showed that EGFR signals (white arrows) are located on the outer membrane of mitochondria. These results above are consistent with other report [23].

### EGF induces EGFR translocation into the mitochondria through endocytosis

EGF is a canonical ligand for triggering EGFR functions; thus we further assessed whether EGF treatment could induce the mitochondrial translocation of EGFR. We found that EGF stimulation substantially increases phosphorylated EGFR in the mitochondria in A549 and H1299 lung cancer cells (Figure 1D; the internal control was presented in Supplementary Figure S1C, implying that the phosphorylated EGFR may translocate from the cell surface to the mitochondria through endocytosis. However, we could not detect the increase of the total EGFR levels in mitochondria upon the EGF treatment, which might be due to the result of endosome-mediated degradation. EGFR endocytosis is critical for EGFR recycle and is also companied with EGFR degradation in the endosomes [24]. Therefore, the endosomal acidification inhibitor, chloroquine [25], was used to alleviate EGFR degradation in the lysosome. We confirmed that mitochondrial localization of both the phosphorylated and total EGFR levels were increased after co-treatment of EGF and chloroquine (Figure 1E). In addition, our data also revealed that although cells were starved for 24 hours, EGFR could still be detected in the mitochondria fraction. And some studies showed that EGFR can also be internalized without EGF treatment [15, 24, 26]. The data supported that EGFR may translocate into the mitochondria through EGFR internalization. Furthermore, the immunofluorescence staining also indicated that the endogenous EGFR can be detected in the mitochondria and EGF treatment induces mitochondrial localization of EGFR in H1299 cells (Figure 1F). To further confirm EGF effects on mitochondrial translocation of EGFR, EGF conjugated with AlexaFluor-488 was used to treat the cells, and the signals of the ligand-receptor complex could not only be shown in the cytosol, but also colocalized with the mitochondria (Figure 1G). These results represent that the EGFR on the cell surface may be internalized and translocated into the mitochondria.

### Mitochondrial EGFR induces mitochondrial fission in NSCLC cells

**Figure 2:**
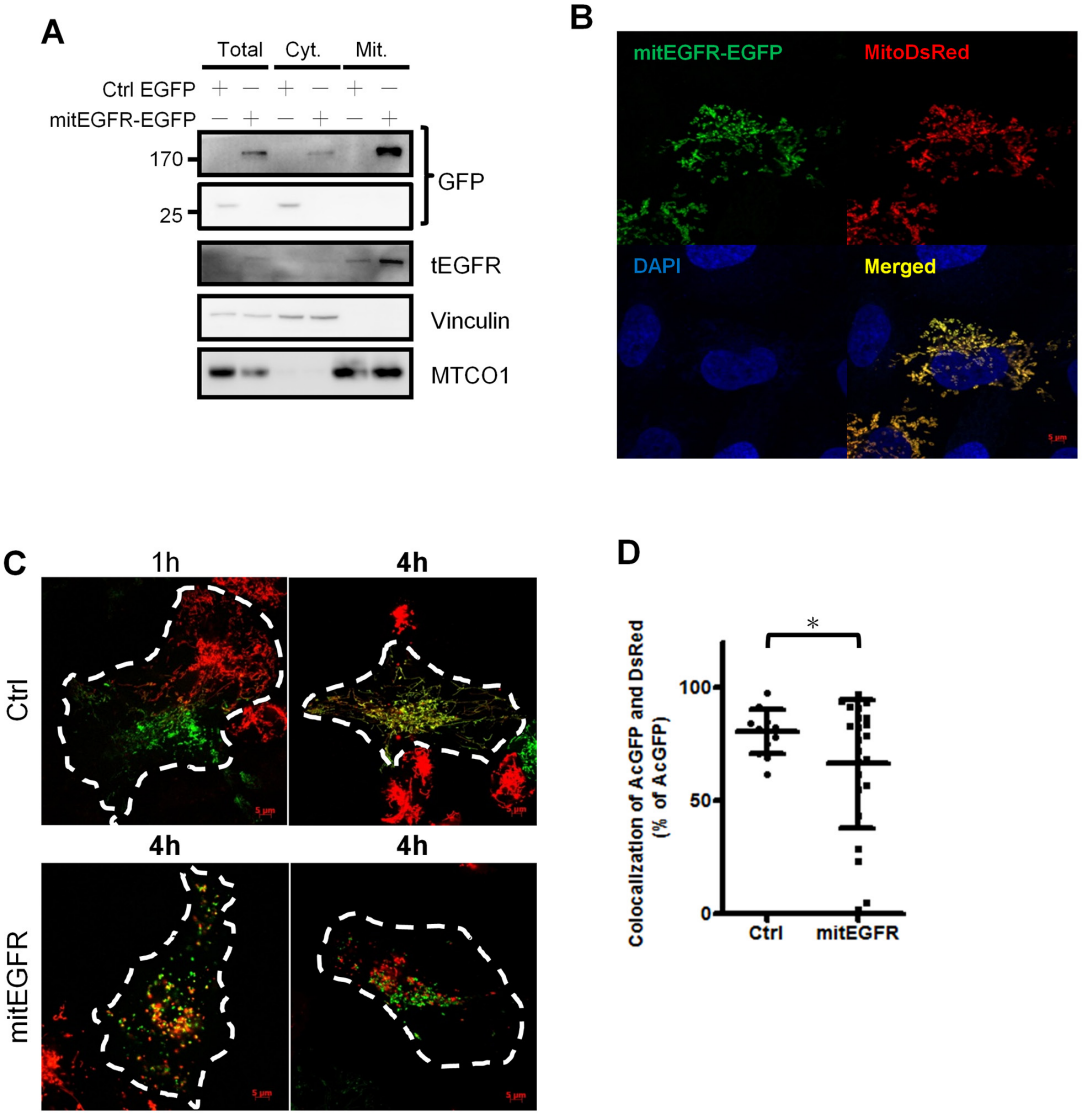
Mitochondrial EGFR inhibits mitochondria fusion by PEG cell fusion assay in NSCLC cells **A.** CL1–0 cells were transfected with EGFP control or mitEGFR-EGFP plasmids, and the expression of mitEGFR-EGFP in the mitochondria was confirmed by immunoblotting. **B.** Immunofluorescence of CL1–0 co-transduced with control EGFP or mitEGFR-EGFP plasmids and pMitoDsRed markers. **C.** Two groups of CL1–0 cells transduced with mitoDsRed or mitoAcGFP were transiently transfected with the control or mitEGFR plasmids. After PEG treatment, the images were obtained after the indicated time. Scale bar: 5 μm. **D.** The percentage of the colocalization was analyzed by MetaMorph software. Mean ± s.d. is shown, *P < 0.05 by Student's *t*-test.

**Figure 3:**
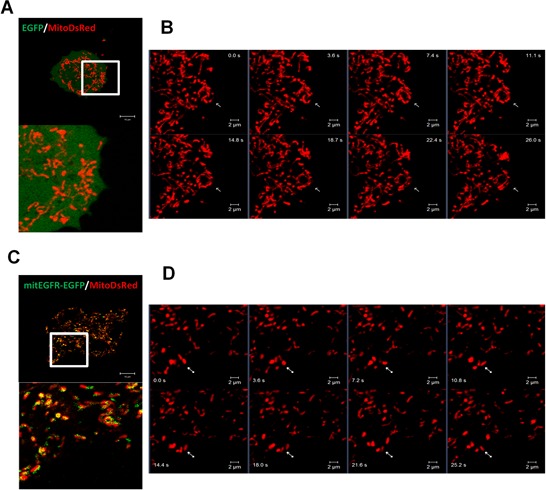
Mitochondrial EGFR disturbs the connection of mitochondria **A–D.** Real-time acquisition of images of the mitochondria dynamics. CL1–0 cells were transfected with the control (A and B) or mitEGFR (C and D) plasmids, and pMitoDsRed plasmids (1/10 of the quantity of control or mitEGFR plasmids), and incubate for 24 h. Time-laspe images were obtained by LSM780 microscope. (A and C) The lower panel is the magnified image of the white square in the upper panel. (B and D) panels are the real-time images of the white square in panel A and C. The images were recorded every 3.6 sec. Scale bar: 2 μm.

To study the effect of EGFR in the mitochondria, mitochondrial-targeting EGFR (mitEGFR) construct was established, and the ectopic overexpression of mitEGFR-EGFP in CL1–0 was shown in Figure 2A. The PK digestion pattern of mitEGFR-GFP was consistent with that of Tom20 and endogenous mitochondrial EGFR (Supplementary Figure S2A). Furthermore, the mitEGFR-EGFP expression was co-localized with mitoDsRed markers (Figure 2B). Interestingly, we found that ectopic mitEGFR-EGFP expression could change the mitochondria morphology (Supplementary Figure S2B). Therefore, we examined the effects of mitEGFR expression on mitochondria dynamics by a polyethylene glycol (PEG) cell fusion assay (Figure 2C and 2D). Two groups of CL1–0 cells which expressed mitoDsRed or mitoAcGFP, respectively, were cocultured and treated with PEG to induce cell fusion. In the upper panel of Figure 2C, control CL1–0 cells started to fuse at 1 h, and complete mitochondria fusion (indicated by colocalizatin of DsRed and AcGFP signals) was visualized 4 h after PEG treatment. Significantly, mitEGFR expression delays mitochondria fusion, and lower levels of mitochondria fusion were observed in mitEGFR-expressing cells (Figure 2C lower panel). The quantitative analysis of the colocalization of AcGFP and DsRed in Figure 2D showed that mitochondria fusion was inhibited in the presence of mitEGFR in CL1–0 cells at 4 h after PEG treatment. Moreover, by using time-lapse microscopy, the mitochondria of CL1–0 cells transduced with EGFP control and mitoDsRed were continuously connected with each other, and could not segregate easily (Figure 3A and 3B). On the other hand, the mitochondria of CL1–0 cells with mitEGFR-EGFP overexpression moved vigorously (Figure 3C and 3D).

**Figure 4:**
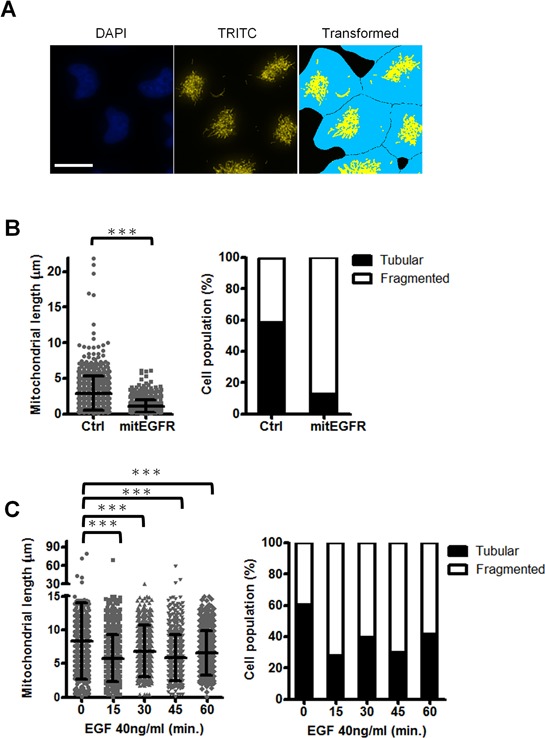
Mitochondrial EGFR induces mitochondrial fission in NSCLC cells **A.** The representative image by the high-content microscopic system was transformed and analyzed by MetaXpress software. Scale bar: 22 μm. **B.** CL1–0 cells transduced with control vectors or mitEGFR plasmids, and mitoDsRed were reseeded in μ-plate 96-well, and stained with CFSE and Hoechst 33342. The images were acquired by the high-content screening system. The mitochondrial length of each groups were analyzed. The images were acquired in 25 different areas in 96-well dish, 6 wells per group. The cell number of the control group and mitEGFR group was 826 and 729, respectively. The right panel showed that the cell populations were categorized into the tubular, and fragmented morphology, according to the mitochondrial length and morphology. **C.** H1299 cells were treated with 40 ng/ml of EGF at 37°C for the indicated time, and the cells were used for analysis of the mitochondrial length with the high-content microscopy. The cell number of 5 groups was 756, 1336, 1205, 1145, and 719, respectively. In all the panels mean ± s.d. is shown, ***P < 0.0001 by Student's *t*-test.

We further evaluated the mitochondria dynamics affected by mitEGFR, and the ImageXpress high-content screening system was used to address the alterations in mitochondrial morphology. The images were acquired and transformed by MetaXpress software to calculate the average length of mitoDsRed per cell (Figure 4A), and the detailed steps of signal transformation and calculation were presented in (Supplementary Figure S3). The left panel of Figure 4B shows that the mitEGFR expression significantly reduces the average length of mitochondria per cell. The mitochondrial morphology is categorized as tubular (representing fusion), and fragmented (representing fission). As shown in the right panel of Figure 4B, 58% and 41% of the cell population were found to harbor tubular and fragmented mitochondria in the control group, consistent with the results in other reports [27]. Significantly, mitEGFR overexpression rendered a higher percentage of cell population to display fragmented mitochondria (Figure 4B). Likewise, we investigated the effect of EGF treatment on the mitochondrial morphology in H1299 cells. We found that the percentage of mitochondrial fragmentation was increased after EGF treatment for the indicated time in both NSCLC cell lines (Figures 4C). The above results showed that EGFR mitochondrial translocation could indeed induce mitochondrial fission.

### MitEGFR expression regulates energy production, cell motility and mitochondria distribution

**Figure 5:**
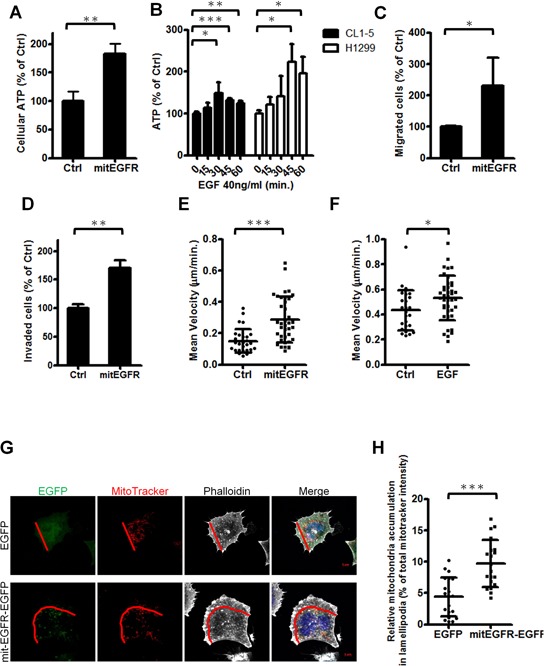
Mitochondrial EGFR enhances energy production, cell motility and alters mitochondrial distribution **A.** CL1–0 cells transfected with the control or mitEGFR plasmids were reseeded in 96-well dishes. After 24 h, the cellular ATP estimation was performed, and the results are presented as the percentage of cellular ATP compared with CL1–0 transfected with control vectors in each group. **B.** After serum starvation for 24 h, CL1–5 and H1299 cells were then treated with 40 ng/ml of EGF for 0, 15, 30, 45, and 60 min in triplicate. The cellular ATP estimation was performed. **C** and **D.** CL1–0 cells transduced with control or mitEGFR plasmids were reseeded for the migration (C) and invasion (D) assay in triplicate. The results are presented as the percentage of migrated or invaded cell number compared with the control group. **E.** CL1–0 cells transfected with EGFP or mitEGFR-EGFP plasmids were reseeded in 12-well dishes for recording images every 20 min with the time-lapse fluorescence microcopy. The speed of the migrated cells is shown, and the cell number of EGFP group and mitEGFR-EGFP group is 29 and 39, respectively. **F.** CL1–5 cells were starved for 24 h. and treated with 40 ng/ml of EGF for recording images every 20 min with the time-lapse fluorescence microcopy. The mean velocity of the migrated cells for 200 min. is shown, and the cell number of untreated group and EGF-treated group is 26 and 38, respectively. **G** and **H.** CL1–0 cells transfected with EGFP or mitEGFR-EGFP and mitoDsRed plasmids were fixed and then staining with Alexafluor 647-labeled phalloidin. The cells were visualized with a LSM700 microscope and the images were analyzed with MetaMorph software. The demonstrated images are shown in panel G (Scale bar: 5 μm.), and the lamellipodia region is defined as the area from the leading edge of a cell to half of the distance to the nucleus, as indicated by the red line. The relative fluorescent intensities in the lamellipodia region of 20 selected cells in each group were normalized to that of the whole cell, and the results are shown in panel H. In all the panels mean ± s.d. is shown, *P < 0.05, **P < 0.001 and ***P < 0.0001 by Student's *t*-test.

Mitochondrial morphology is correlated with crucial cellular behaviors [28, 29], and mitochondrial dynamics is also linked to the balance between energy demand and nutrient supply [9]. Thus, we evaluated the influence of mitEGFR on cellular ATP productive activity. The results showed that mitEGFR could significantly enhance cellular ATP production (Figure 5A). We then examined the effects of endogenous mitochondrial EGFR after EGF stimulation on ATP production. The ATP production in the EGF-treated cells was increased by up to 1.5~2-fold comparing to the untreated control in CL1–5 and H1299 cells (Figure 5B). Regardless of the low expression of EGFR in the mitochondria in CL1–0 cells, the ATP production was also increased slightly after EGF stimulation (Supplementary Figure S4).

The mitochondria dynamics is related to cell motility [12], and thus the effects of mitochondrial EGFR expression on cell migration ability were evaluated by the migration assay, the invasion assay and the single-cell motility tracking assay. The results revealed that cell migratory ability was increased about two-fold in the presence of mitEGFR (Figure 5C), and the invasiveness of mitEGFR-expressing CL1–0 cells was also enhanced about 1.5-fold (Figure 5D). Moreover, the speed of migrated cells was significantly increased in the mitEGFR-expressing cells (Figure 5E; the routes were presented in Supplementary Figure S5A). We also studied the effects of endogenous EGFR on cell motility. EGF stimulation increased the mean velocity of CL1–5 cells in 200 min. (Figure 5F), and the routes and the distance to the origin of CL1–5 cells treated with EGF were represented in Supplementary Figure S5B and S5C. Therefore, mitochondrial EGFR enhances ATP production and cell motility *in vitro*.

The distribution of mitochondria in cells is correlated to cell motility [12, 30]. We evaluated the impact of mitEGFR on the subcellular mitochondria distribution. The actin filaments stained by phalloidin indicated the direction of cell movement, and the signals of MitoTracker in the front of cell movement, lamellipodia, were analyzed. The percentage of mitochondria in the lamellipodia region was calculated (Figure 5G), and as a result, the subcellular distribution of mitochondria was increased in the lamellipodia area after the mitEGFR overexpression (Figure 5H).

### EGFR in the mitochondria enhances mitochondria fission independent of its phosphorylation status

**Figure 6:**
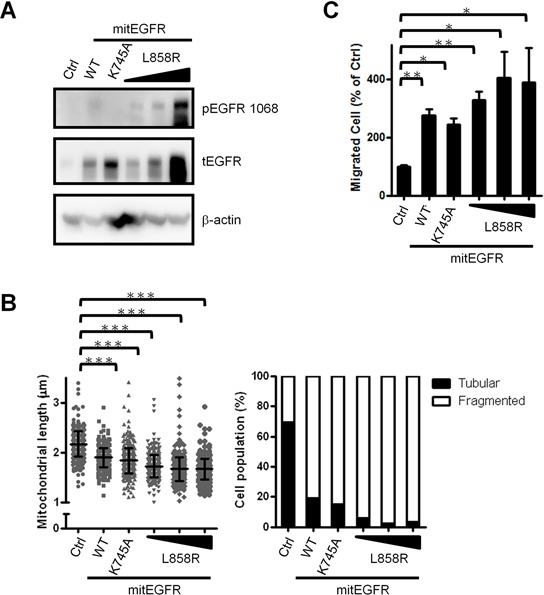
Mitochondrial EGFR induces mitochondria fission independent of its phosphorylation status **A.** CL1–0 cells were transduced with control or mitEGFR mutant plasmids. And the cells were subjected into immunoblotting for confirming the expression and phosphorylation of EGFR. **B.** Mitochondria length per cell was estimated by the high-content microscopic system in CL1–0 transducd with mitEGFR mutant constructs. The right panel showed that the cell populations were categorized into the tubular and fragmented morphology, according to the mitochondrial length and morphology. The cell number of 6 groups was 455, 425, 427, 428, 451 and 379 respectively. **C.** CL1–0 cells were co-transduced with control or mitEGFR mutant plasmids, and then reseeded in the transwells in triplicate. After 22 h, migrated cells were quantified, and the relative percentage of migrated cells in each group to that in the control group is presented. In all the panels mean ± s.d. is shown, *P < 0.05, **P < 0.001 and ***P < 0.0001 by Student's *t*-test.

In NSCLC, EGFR phosphorylation status is correlated to therapeutic prognosis and relapse [31]. Next, we investigated whether EGFR phosphorylation status contributes to the regulation of mitochondria dynamics. The expression level and the phosphorylation status in CL1–0 cells transduced with mit-EGFR mutants, including a constitutively active mutant, L858R, and a kinase dead mutant, K745A [26] were confirmed (Figure 6A). Next, the cellular mitochondria length was evaluated, and we found that all of the mitEGFR mutants enhance the mitochondria fission (Figure 6B) and the cellular motility (Figure 6C), compared to the control group in CL1–0 cells. Therefore, EGFR in the mitochondria promotes mitochondria fission and cell motility in NSCLC, independent of its phosphorylation status.

### EGFR regulates mitochondrial dynamics through interacting with Mfn1

**Figure 7:**
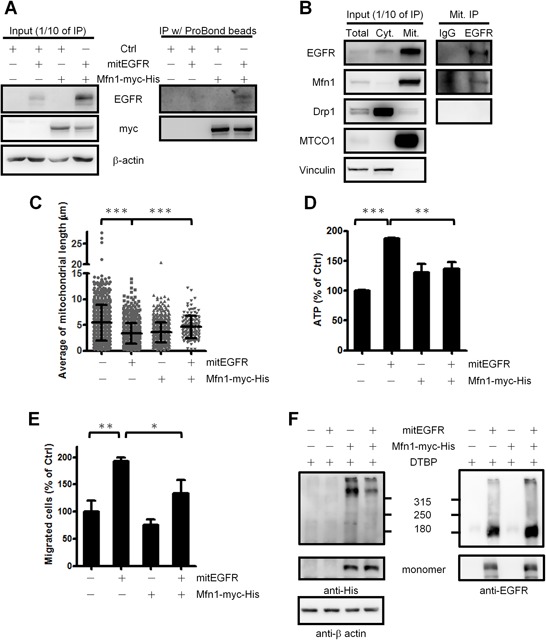
EGFR regulates mitochondrial dynamics through interacting with Mfn1 functions **A.** Co-immunoprecipitaion assay of mitEGFR and Mfn1-myc-His. The lysates of CL1–0 cells co-tranduced with control, mitEGFR or Mfn1-myc-His were incubated with ProBond nickel-chelating resin at 4°C for 5 h, and then the mixtures were subjected to immunoblotting. **B.** The mitochondria of H1299 cells were subtracted, and lysed with IP lysis buffer. The mitochondrial fractions were incubated with anti-EGFR IP-specific or mouse IgG control antibodies at 4°C for 5 h, and then protein A beads were added for 1 h. The mixtures were subjected to Western blotting. **C.** CL1–0 cells co-transduced with control, mitEGFR or Mfn1 plasmids, as indicated, were reseeded for the analysis of the mitochondrial length by high-content microscopy. The cell numbers of these four groups were 848, 662, 711 and 118, respectively. Mean ± s.d. is shown. The expression of these plasmids was confirmed by immunoblotting (Supplementary Figure S6C). **D.** CL1–0 cells were co-transduced with the plasmids, as indicated, and reseeded in 96-well dishes in triplicate. ATP production was measured, and the relative percentage of ATP in each group to that in the control group is presented as the Mean ± s.d.. **E.** CL1–0 cells were co-transduced with control, mitEGFR or Mfn1 plasmids, and then reseeded in the transwells in triplicate. After 22 h, migrated cells were quantified, and the relative percentage of migrated cells in each group to that in the control group is presented as the Mean ± s.d., *P < 0.05, **P < 0.001 and **P < 0.001 by Student's *t*-test. **F.** CL1–0 cells co-transduced with the plasmids as indicated were treated with DTPB 5 mM for 30 min, and the cells were subjected into the gradient SDS-PAGE electrophoresis. In the upper panel, the indicated bands represent Mfn1-containing protein complex. And the lower panel showed the monomer of Mfn1 and mitEGFR expression. The β-actin expression serves as an internal control.

Mfn1 [32], optical atrophy protein 1 (OPA1) and dynamin-related protein 1 (Drp1) [33] orchestrate the dynamics of mitochondrial fusion and fission [6, 34]. We explored if mitEGFR affected the expression levels of these key components in mitochondrial dynamics. As a result, mitEGFR overexpression did not alter the protein expression levels as well as the activation status of Drp1 (indicated by the phosphorylation level of Drp1) (Supplementary Figure S6A). From the immunoprecipitation experiments, we found that the ectopic mitEGFR interacted with Mfn1 (Figure 7A), but not Drp1 (Supplementary Figure S6B). Moreover, the endogenous EGFR in the mitochondria was associated with Mfn1, but not with Drp1 (Figure 7B).

We next determined whether EGFR in mitochondria affects mitochondria dynamics through regulating Mfn1. The Mfn1 reintroduction could rescue the shortened mitochondrial length caused by mitEGFR expression (Figure 7C). Moreover, Mfn1 co-expression significantly alleviated the effects of mitEGFR-mediated higher ATP production and cell migration (Figure 7D and 7E). These results suggest that Mfn1 plays a role in the mitEGFR-mediated regulations in mitochondrial dynamics.

We then investigated how mitochondrial EGFR regulates Mfn1. Mfn1 is located on the mitochondrial outer membrane, and exerts its function to help mitochondria fusion through polymerization [35, 36]. Accordingly, we investigated whether the mitochondrial EGFR disturbs Mfn1 polymerization to increase the mitochondria fragmentation by the chemical crosslinking. (Figure 7F) shows that the presence of mitEGFR evidently decreased the amounts of Mfn1 in the protein complex, comparing to the cells with Mfn1 expression only. The results support that mitochondrial EGFR may associate with Mfn1 to reduce its polymerization, which then impede the Mfn1-mediated activity in promoting mitochondria fusion.

### Mitochondrial EGFR expression increases cancer metastasis *in vivo* and is associated with lymph node metastasis in NSCLC

**Figure 8:**
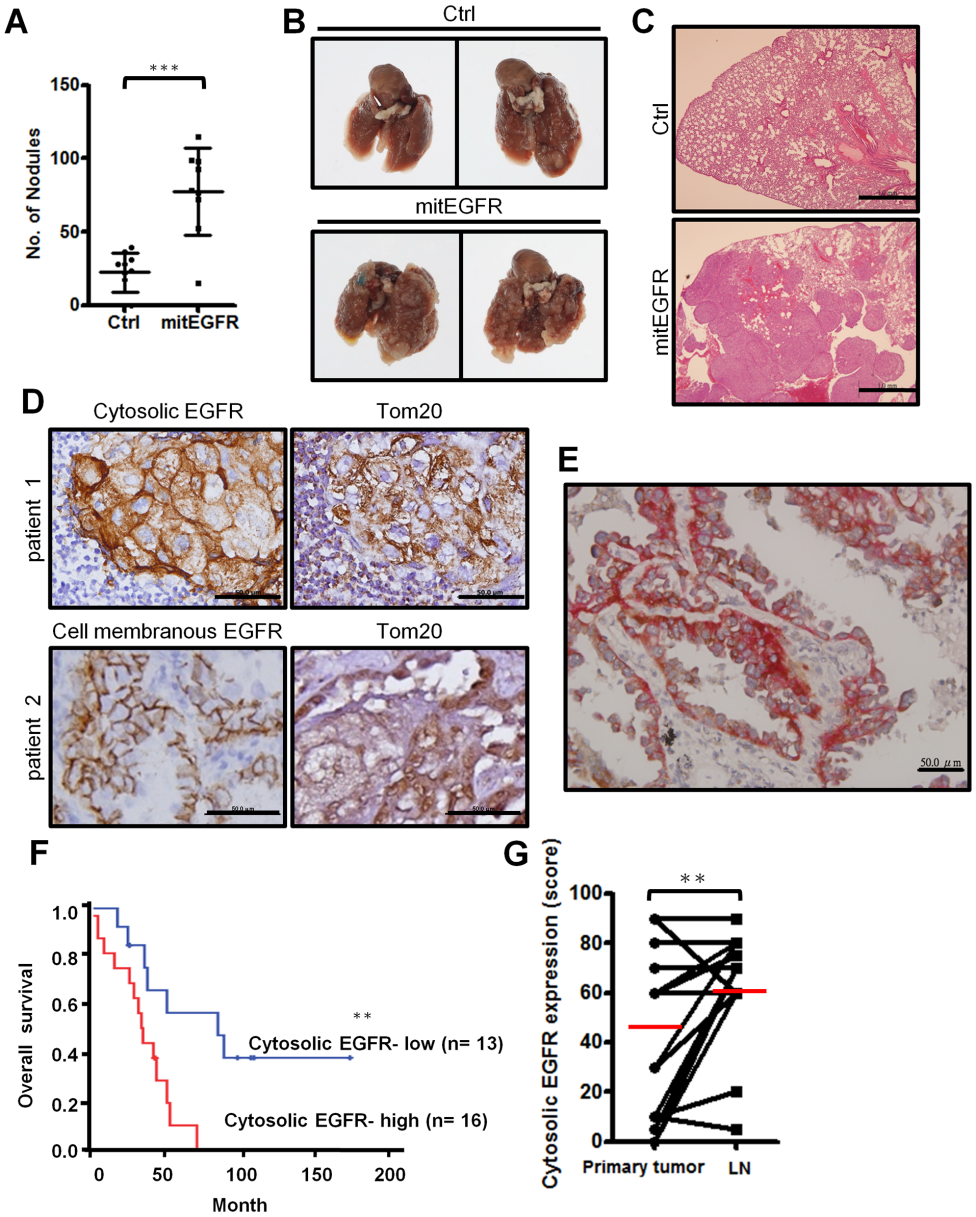
*In vivo* metastasis model of mitEGFR and the clinical correlations of cytosolic EGFR expression in NSCLC **A–C.** Effects of mitEGFR overexpression on metastasis *in vivo*. NOD-SCID mice were injected with control or mitEGFR-expressing CL1–5 cells intravenously. Quantitative evaluation of lung metastatic nodules 3 weeks after tail-vein injection was presented in panel A as the Mean ± s.d., ***P < 0.0001 by Student's *t*-test. The number of mice injected with CL1–5/Ctrl or CL1–5/mitEGFR cells was 10 or 9, respectively. (B & C) The representative lungs and sections by H&E stain of mice intravenously injected with CL1–5/Ctrl (upper panel) or CL1–5/mitEGFR (lower panel) cells. Scale bar: 1 mm. **D.** Immunohistochemical analysis of EGFR and Tom20 expression. The upper left is cytosolic EGFR expression, and the lower left is cell-surface EGFR expression. The right panels are Tom20 expression of the serial sections from the left panels. Scale bar: 50 μm. **E.** The representative image by the IHC double staining of Tom20 and EGFR. The brown color represents Tom20 signals (HRP), and the red color represents EGFR signals (AP). Scale bar: 50 μm. **F.** Kaplan-Meier plots of overall survival in 29 NSCLC patients in high- and low-risk group based on cytosolic EGFR expression levels. *P*-values were obtained from log-rank tests. *P* = 0.0078. **G.** The cytosolic expressions in the primary tumor site and in the paired lymph node were scored. Red bars represent mean, and *P* = 0.0142.

Our studies showed that mitochondrial EGFR can regulate mitochondria dynamics and cell motility *in vitro*. To study whether mitochondrial EGFR enhances metastasis *in vivo*, we injected control or mitEGFR-expressing CL1–5 cells intravenously into non-obese diabetic-severe combined immunodeficiency (NOD-SCID) mice. After 21 days, the quantitative analysis in Figure 8A showed that mice injected with CL1–5/mitEGFR cells developed more pulmonary nodules than those injected with control cells. The gross morphology of the lungs and the H&E staining of the lung sections (Figure 8B and 8C) showed severe lung tumor growth in the mice injected with CL1–5/mitEGFR cells, compared to the mice injected with CL1–5/Ctrl cells.

Next we examined the clinical significance of mitochondrial EGFR expressions in paired samples of primary lung tumors and metastatic lymph nodes of 29 NSCLC patients (Supplementary Table S2). Cytosolic and cell membranous EGFR, and the mitochondrial marker, Tom20, were evaluated by immunohistochemical staining in serial sections of the samples, and the representative images are demonstrated in Figure 8D. Interestingly, the signals of cytosolic EGFR shared the similar compartment to the localization of the mitochondrial marker, Tom20, and this implied that the majority of cytosolic EGFR was located in the mitochondria, and the colocalization of EGFR and Tom20 was checked by IHC double staining (Figure 8E). Kaplan-Meier analysis showed that high levels of cytosolic EGFR expression were significantly associated with poor overall survival (*P* = 0.0078; Figure 8F), while cell membranous EGFR expression was not related to the survival of NSCLC patients (data not shown). The cytosolic EGFR expression levels in the lymph node are higher than its expression in the primary tumor (*P* = 0.0142, Figure 8G). Cox proportional hazard regression analysis with a stepwise selection model also demonstrated that the overall survival of this cohort was correlated with cytosolic EGFR expression levels (H*R* = 1.016) (Supplementary Table S3). The results from *in vivo* animal model and the histological analysis coincide with the *in vitro* results, indicating that mitochondrial EGFR may influence mitochondrial behavior, cell motility and clinical outcomes.

## DISCUSSION

**Figure 9:**
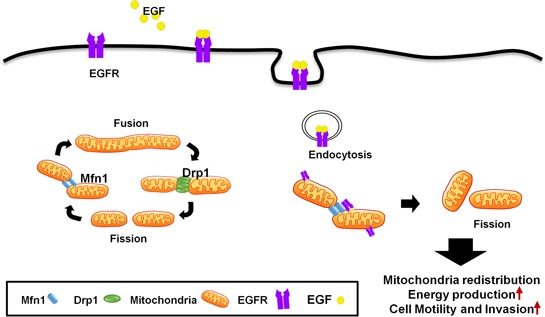
A schematic model of mitochondrial dynamic regulated by EGFR EGFR translocates from the cell membrane into the mitochondria, and induces mitochondrial fission through inhibition of Mfn1. Therefore, mitochondrial translocation of EGFR promotes ATP production, cell motive ability and mitochondrial redistribution.

EGFR is not only a classic membranous receptor, but also a multifunctional regulator in other subcellular organelles [31]. We found a novel mechanism that EGFR translocates into the outer membrane of the mitochondria through endocytosis, and is involved in regulating mitochondria dynamics (Figure 9). Mitochondrial EGFR-induced fission is correlated to energy production and mitochondrial redistribution to the lamellipodia area, resulting in the increasing cell motility *in vitro* and metastasis *in vivo*. We also showed that EGFR could interact with Mfn1 and interfere the polymerization of Mfn1. Interestingly, the cytosolic EGFR expression in the lymph node is higher than the expression in the paired sections of primary tumor. The EGFR expression in cytosolic regions is associated with poor clinical outcome in NSCLC patients. These *in vitro*, *in vivo* and clinical findings support that mitochondrial EGFR can promote mitochondrial fission by disturbing Mfn1 polymerization, re-distribute mitochondria, and enhance ATP production to provide sufficient energy for cellular movement.

In a canonical pathway, EGFR induced by EGF will be phosphorylated and activate downstream effectors, and at the same time, EGFR is internalized for degradation by ubiquitination in the endosome/lysosome or recycling back to the cell membrane [37]. Thus, we found that the endosomal acidification inhibitor, chloroquine, can enhance the presence of EGFR in the mitochondria by preventing the degradation of EGFR in the endosomes (Figure 1E). In addition, our data revealed that although cells were under serum starvation for 24 hours, EGFR is still detected in the mitochondria. With the low concentration of EGF, EGFR can still be internalized for recycling or degradation [24]. By combining previous findings [23], we thought that the translocation of EGFR into the mitochondria might be through endocytosis. A similar finding revealed that the nuclear translocation of EGFR was also through endocytosis [18]. Scientists showed that a cell membrane glycoprotein, MUC1, can regulate endocytosis and nuclear translocation of EGFR, even without EGF stimulation [38]. Moreover, nuclear trafficking of EGFR was through microtubule and syntaxin-6 mediated endocytosis [19]. It would appear that these findings imply the mechanisms of EGFR-related mitochondrial trafficking. However, how endocytotic EGFR trafficks to different organelles require further study.

Mitochondrial dynamics are correlated to many mitochondrial functions and essential to cellular fates, which are linked to cell death, development, aging and cancers [6, 7]. Mitochondria are usually distributed throughout the body and neurites of the neuronal cells by microtubule-mediated mitochondria transport, and the balance of mitochondrial fusion and fission is critical for neuronal functions [5, 7]. Fission-deficient mitochondria aggregates in cells and represses mitochondrial distribution in neurites, and then leads to loss of ATP supply, disturbance of Ca^2+^ homeostasis, oxidative stress, and finally neurodegenerative diseases [10, 39]. In cardiac diseases, mitochondria dynamics is also correlated to calcium homeostasis, apoptosis, vascular smooth muscle cell proliferation [3], and evidences showed that decreasing mitochondrial fission due to the mutation of mitochondrial fission gene *Dnm1* induces cardiomyotrophy [40]. Therefore, mitochondrial dynamics is critical for the maintenance of cellular life.

Notably, mitochondrial dynamics also play roles in cell motility [29]. In breast cancer, highly malignant cells usually have separated mitochondria, and manipulation of mitochondrial dynamics results in the alteration of cell motility [12]. Furthermore, the distribution of mitochondria in cells also plays a role on lamellipodia formation [12, 41]. The localization of mitochondria in the leading edge of migrating epithelial cells correlated with faster migration speed and increased directional persistence [30], suggesting that mitochondria may quickly provide sufficient energy at the area which eagerly needs [3]. Insufficient ATP supply due to mitochondrial dysfunctions inhibiting cancer cell motility [42]. On the other hand, we found that mitEGFR enhances the mitochondria fission and cancer cell motility, independent of its phosphorylation status. Several reports revealed that EGFR has other functions, including maintenance of cellular glucose level [43] and the initiation of autophagy [26], independent of its kinase activity. Interestingly, in prostate cancer cells, researchers found that EGFR induced mitochondrial fusion through upregulation of OPA1 and involve in *de novo* synthesis of the fatty acid, palmitate [44]. By using the prostate cancer cell line, PC3 cells, we also found that EGF treatment indeed induced mitochondrial elongation (Supplementary Figure S7, *P* < 0.0001). However, we did not observe the protein level of OPA1 changed under the overexpression of mitEGFR in NSCLC cells (Supplementary Figure S6A). Therefore, the contrary molecular mechanism EGFR engaged in the mitochondria may come from the different cellular context and the behaviors of each type of tissue or organ. However, our findings are consistent with theirs, which indicated that mitochondrial EGFR plays an important role on cellular metabolism.

The key components of mitochondrial dynamics, including Drp1, Fis1, Mfn1, Mfn2, and OPA1, orchestrate mitochondrial morphology to deal with the contingency or stress [34]. Our results showed that EGFR can interact with Mfn1, rather than Drp1, to induce mitochondrial fragmentation. Mfn1 has a GTPase domain, two transmembrane segments (TM), and two hydrophobic heptad repeats (HR), and Mfn1 polymerizes *in trans* through HR2 domain by assembling an antiparallel-coiled coil [35]. We found that mitochondrial EGFR can interact with Mfn1, interfere with Mfn1 polymerization, and then inhibit mitochondrial elongation. After reintroduction of Mfn1, mitochondria fragmentation and cell motility was inhibited. However, the detailed mechanism of how EGFR disturbs Mfn1 polymerization needs to be further investigated, as it may be through the inhibition of Mfn1 GTPase activity or masking HR2 domain, which is critical for polymerization [35].

EGFR dysregulation is correlated to the progression of NSCLC [14], and it is intriguing that other than its RTK functions, EGFR in different cellular localization may also participate in the processes of oncogenesis. In this study, EGFR in the cytosol is analyzed by IHC staining for representing the biological significance of mitochondrial EGFR, and we found that cytosolic EGFR expression is relevant to the overall survival of NSCLC patients, but the correlation of cell surface EGFR expression and the survival is not significant (data not shown). Other researchers found that nuclear EGFR cooperates with STAT3 to regulate iNOS expression, and its nuclear expression is positively correlated to iNOS expression, a prognostic marker for breast cancer [45]. Nevertheless, in metastatic renal cell carcinoma (RCC), membranous EGFR is highly present in the tumor sample and higher cytosolic EGFR expression was in the paired normal renal tissue [46]. These observations suggested that multiple roles of EGFR may depend on its cellular localization, and the differential microenvironment that came from different cell-types.

In conclusion, mitochondrial EGFR translocation may cause mitochondrial fission, alters mitochondrial subcellular distribution and energy production as well as metastasis in NSCLC. The underlying mechanisms are related to the interaction of EGFR and Mfn1, and the decreasing Mfn1 polymerization. These findings provide a multifaceted role of EGFR in lung cancer progression and metastasis.

## MATERIALS AND METHODS

### Cell lines

The lung cancer cell lines CL1–0 and CL1–5 were derived from *in vitro* transwell and *in vivo* metastasis selection as previously described [21]. A549, H1299, H3255, HCC827 were obtained from American Type Culture Collection (ATCC) (VA, USA). PC9IR was a kindly gift from Dr. Sung-Liang Yu and Dr. Jen-Yi Lee (National Taiwan University). The CL1–0, CL1–5, PC9IR and H3255 cells were maintained in RPMI medium supplemented with 10% fetal bovine serum. The A549 and H1299 cells were maintained in DMEM with 10% fetal bovine serum. All of the cell lines were incubated in the humidified chamber with 5% CO_2_ at 37°C.

### Mitochondrial fractionation

Qproteome mitochondria isolation kit (Qiagen, Venlo, Netherlands) was used for mitochondrial fractionation, and the procedures followed the manufacturer's handbook.

### iTRAQ (isobaric tag for relative and absolute quantitation) on mitochondria proteome

Briefly, the mitochondria of lung cancer cell lines with different invasion abilities (CL1–0 and CL1–5) were isolated and subjected to gel-assisted digestion with trypsin [47]. The resulting peptides from CL1–0 were labeled with iTRAQ_114_, iTRAQ_116_, while peptides from CL1–5 were labeled with iTRAQ_115_, and iTRAQ_117_, respectively. After reaction, the labeled peptides were combined and separated by strong cation exchange (SCX) fractionation (PolyLC, Columbia, MD). Each SCX fraction was analyzed by Waters Synapt HDMS coupled with a nanoACQUITY Ultra Performance LC^TM^ system (Waters Corp., Milford, MA) using a 20-mm × 180-mm trap column and separated by 200-mm × 75 mm Waters1 ACQUITY 1.7 mm BEH C18 column. The data analysis for the iTRAQ experiments was performed with the developed software Multi-Q [48].

### Antibodies

The primary antibodies used for immunoblot analysis and immunohistochemical staining were rabbit anti-EGFR, mouse anti-vinculin, mouse anti-Tom23, goat anti-Tim23, rabbit anti-Mfn1, mouse anti-Drp1 (all above from Santa Cruz Biotechnology, CA, USA), mouse anti-MTCO1 (Abcam, Cambridge, UK), rabbit anti-phospho-EGFR (Tyr1068), rabbit anti- phospho-Drp1 (Ser637) (all above from Cell Signaling Technology, Inc., MA, USA), mouse anti-OPA1 (BD Biosciences, NJ, USA) and mouse anti-myc, mouse anti-Flag, mouse anti-β actin antibody (all above from Sigma-Aldrich, Inc., MO, USA). The primary antibodies used for immnofluorescence staining were rabbit anti-EGFR (Abcam, Cambridge, UK) antibody.

### Western blot analysis

Cell lysates were subjected to SDS-polyacrylamide gel electrophoresis before transfer to PVDF membrane (ThermoFisher Scientific Inc., MA, USA). Primary antibodies were used according to the conditions recommended by the manufacturer. Bound antibody was detected using the Western Lightning Plus ECL reagents (PerkinElmer, MA, USA). Chemiluminescent signals were captured by BioSpectrum imaging system (UVP, CA, USA).

### Electron microscopy

H1299 cells were seeded onto sterile ACLAR embedding films (Electron Microscopy Sciences, PA, USA), cultured for 24 h, starved for 24 h, and stimulated with 40 ng/ml of EGF for 10 min. Cells were fixed with the solution including 2.5% glutaraldehyde and 4% paraformaldehyde in PBS at 4°C overnight. After washing with 0.1 M sodium carcoldylate buffer 3 times, 10 min/time, the cells were then fixed with 1% OsO4 diluted by 0.1 M carcodylate buffer for 2 h on ice. After washing with 0.1 M carcodylate buffer for 3 times, 10 min/time, the cells were then subjected to serial ethanol dehydration (30%, 45%, 60%, 75%, 90%, 100%), 30 min each step, Then the cells were embedded in Spurrs' resin and polymerized at 70°C for 16 h. The samples were then sectioned into ultrathin sections in 90 nm thickness, and then set for immunostaining with anti-EGFR antibodies (Abcam, Cambridge, UK). The secondary antibodies conjugated with 18 nm gold particles were used for detection. The samples were observed using JOEL JEM-1200EX (EM Lab Services, KS, USA).

### Immunofluorescence and confocal microscopy

To determine distribution of mitochondria, cells were loaded with 200nM MitoTracker Red (Life Technologies Corporation, NY, USA) for 15 min. to stain mitochondria. Then cells on coverslips were fixed with 4% paraformaldehyde in phosphate-buffered saline, permeablized with 0.1% Triton X-100, blocked with 3% bovine serum albumin, and then incubated with primary antibodies and fluorescein isothiocyanate-conjugated secondary antibodies. Cells were visualized with a confocal microscope (LSM 700; Carl Zeiss, Jena, Germany) and processed using Zen 2009 software (Carl Zeiss, Jena, Germany). For lamellipodia staining, cells were fixed and stained with Alexa fluor 647-labeled phalloidin. Lamellipodia were identified as a thick stretch with filopodia of perpendicular actin stain at the peripheral edge of the cell as imaged by the phalloidin stain [12]. The lamellipodia region was defined as the area from the leading edge of a cell to half of the distance to the nucleus, and the ratio of the mitochondria in the lamellipodia region v.s. in the total region was analyzed by MetaMorph software (MetaMorph Inc., TN, USA).

### Drug treatment

EGF was purchased from R&D Systems, Inc. (MN, USA), DTBP (Dimethyl 3,3′-dithiobispropionimidate•2HCl) was purchased from ThermoFisher Scientific Inc. (MA, USA), and chloroquine was purchased from Sigma-Aldrich, Inc. (MO, USA). EGF biotinylated, conjugated with Alexa Fluor 488 streptavidin was obtained from Life Technologies Corporation (NY, USA). Stock solutions of EGF and chloroquine were prepared in PBS buffer and stored at −20°C. The compounds were diluted in fresh media before each experiment. Cells were seeded in 150-mm dishes at 80% confluence for serum starvation for 24 h. The cells were pretreated with 5 mM concentration of chloroquine for 3 hours, and then treated with 40 ng/ml concentration of EGF for the indicated time. DTBP working solution 5 mM was prepared in PBS buffer and stored at 4°C. Protein crosslinking was performed by treating the cells with DTBP at 37°C for 30 min.

### Constructs and transfection

pMitoDsRed and pMitoAcGFP plasmids were purchased from Clontech Laboratories, Inc. (CA, USA). The membrane-targeting sequence of EGFR (1–72 nt.) was replaced with the mitochondrial-targeting sequence from subunit VIII of human cytochrome c oxidase, and mitEGFR wild-type and mutants, L858R and K745A, were subcloned into pCDNA3.1 and pEGFP-N1 plasmids. pCDNA3.1/myc-His-Mfn1 and pCDNA3-HA-Drp1 plasmids are kindly gifts from Dr. Joanne Jeou-Yuan Chen [49] and Dr. Alex van der Bliek [50], respectively. All transfection experiments were performed with Lipofectamine 2000 reagents (Life Technologies Corporation, NY, USA) in accordance with the manufacturer's protocol.

### Analysis of mitochondrial length by high-content microscopy

The cells were transfected with the indicated plasmids for 24 h, and then reseeded into the μ-plate 96-well (ibidi, Martinsried, Germany). Before acquisition of the images, cells were stained with Hoechst 33342 dye and CellTrace CFSE dye (Life Technologies Corporation, NY, USA), and then fixed with 4% paraformaldehyde in PBS. The images were acquired by ImageXpress Micro XL System (Molecular Devices, CA, USA). The images were analyzed by MetaXpress Image Acquisition and Analysis Software (Molecular Devices, CA, USA), and the steps of the module were illustrated in Supplementary Figure S3. The mitochondrial signals were analyzed for the average of mitochondrial length per cell.

### Estimation of cellular ATP production

Adenosine 5′-triphosphate (ATP) bioluminescent somatic cell assay kit (Sigma-Aldrich, Inc., MO, USA) was performed for estimation of cellular ATP production. After the transfection with the indicated plasmids, the cells were reseeded in 96-well dishes in the same cell number. After 24 hours, measurement of cellular ATP production was performed according to the manufactured information.

### Migration assay, invasion assay and the single-cell tracking assay

Migration or invasion assays were performed using 24-well transwell inserts (8-μm pore size; BD Falcon, NJ, USA). 2.5 × 10^4^ cells were suspended in 10% NuSerum-containg media (Gibco BRI, NY, USA), seeded in the chamber without or with 6 μg of Matrigel, respectively, and cultured for 20 h. Cells that migrated from top to bottom of the chamber were fixed with methanol and stained with a 50-μg ml^−1^ solution of propidium iodine (Sigma-Aldrich Inc., MO, USA). The propidium iodine-positive signal was quantified using the Analytical Imaging Station software package. Each sample was assayed in triplicate.

Real-time cell migration was recorded by time-lapse confocal microscopy. Briefly, the cells transfected with the indicated plasmid were plated on 12-well dishes. Fluorescence and phase-contrast images were recorded every 20 min for 24 h using Leica DMI 6000B (Leica Microsystems GmbH, Wetzler, Germany). And the images were processed with MetaMorph software.

### Immunoprecipitation assay

For the detection of EGFR-Mfn1 complex, mitochondria fractions were obtained by Qproteome mitochondria isolation kit, and then subtracted in IP lysis buffer [20 mM Tris (pH 8.0), 150 mM NaCl, 100 μM Na_3_VO_4_, 50 mM NaF, 30 mM Na pyrophosphate, and 0.5% NP-40] containing cocktail of protease inhibitor (Roche Diagnostics, Basel, Switzerland). Mitochondrial lysates were incubated with EGFR IP-specific mouse monoclonal antibody (Cell Signaling, MA, USA) or control mouse IgG antibody (Santa Cruz Biotechnology, CA, USA) for 5 h at 4°C. Protein A-Sepharose beads (GE Healthcare, Little Chalfont, UK) were added to the immunoprecipitates and incubated at 4°C for 1 h. The beads were collected by centrifugation at 1,000 g for 3 min and washed 3 times in IP lysis buffer. Proteins were eluted with the SDS protein sample buffer before Western blotting with specific antibodies against Mfn1 and EGFR (Santa Cruz, CA, USA).

### Viruses and transduction

MitEGFR construct was subcloned into pAS2neo lentiviral vectors. Lentivirus was prepared in accordance with standard protocols. In brief, HEK293T cells were co-transfected with pAS2neo-mitEGFR, pCMVΔR8.91, and pMD.G. Virus-containing medium was collected at 24-, 48-, and 72-h post-transfection, and then concentrated. To generate the stably transduced clones, cells were infected with lentivirus in medium containing polybrene (8 μg/ ml). At 24 h after infection, cells were treated with 400 μg/ ml G418 for the selection of a pool of antibiotic-resistant clones.

### *In vivo* metastasis assay

For the *in vivo* tail vein metastasis assay, a single-cell suspension containing 10^6^ CL1–5 cells in 0.1 ml of Hank's Balanced Salt Solution (HBSS) was injected into the lateral tail veins of ten 6-week-old NOD-SCID male mice each group (supplied by BioLASCO Taiwan Co., Ltd, Taiwan). After 21 days, the mice were sacrificed and the lungs were examined for metastasis. The lungs were fixed in 10% formalin, and the number of lung tumor nodules was counted under a dissecting microscope. The experimental procedures were conducted and approved in accordance with the regulations of Academia Sinica Institution Animal Care and Utilization Committee (Taiwan).

### Clinical lung cancer samples and immunohistochemistry

The lung cancer specimens from the primary tumor site and the lymph nodes were from 29 consecutive patients who had undergone surgical resection of NSCLC at the National Taiwan University Hospital, and were analyzed for the expression of membranous and cytosolic EGFR, and Tom20 (serial sections). Sections were fixed in formalin and embedded in paraffin. The primary antibodies against EGFR and Tom20 were obtained from Santa Cruz Technology (CA, USA). PBS without primary antibodies was applied as the negative control. The immnunohistochemical results were scored and classified into two groups according to the average staining intensity and area. Group of the low expression corresponded to a positive staining of < 50% of the membranous or cytosolic part of the tissue section, and group of the high expression corresponded to a positive staining of > 50% of the two parts of the tissue section. The immunostaining results were assessed and scored independently by two pathologists. IHC double staining was performed with MultiView (mouse-HRP/rabbit-AP) IHC kit (Enzo Life Sciences, Inc., NY, USA), and the procedures followed the manufacturer's handbook.

### Statistical analysis

Factors with difference between two groups (High and low cytosolic EGFR expression) were assessed by Student's *t*-test and Fisher's exact test. Multivariate Cox proportional hazard regression analysis was used to evaluate the associations between the abundance of the cytosolic EGFR with patient survival. The factor of tumour stage was considered. Kalpan-Meier plot and log-rank test were also performed. All analyses were performed with SAS version 9.1 software (SAS Institute Inc.). *P*-value < 0.05 were considered to indicate statistical significance.

## SUPPLEMENTARY FIGURES AND TABLES


