# Supplementary material for: Mitochondrial translocation of EGFR regulates mitochondria dynamics and promotes metastasis in NSCLC
Source: Oncotarget. 2015 Oct 19;6(35):37349–66. doi: 10.18632/oncotarget.5736 (PMC4741934; doi:10.18632/oncotarget.5736)
Supplement: Supplementary file 1 [file oncotarget-06-37349-s001.pdf]

SUPPLEMENTARY FIGURES AND TABLES

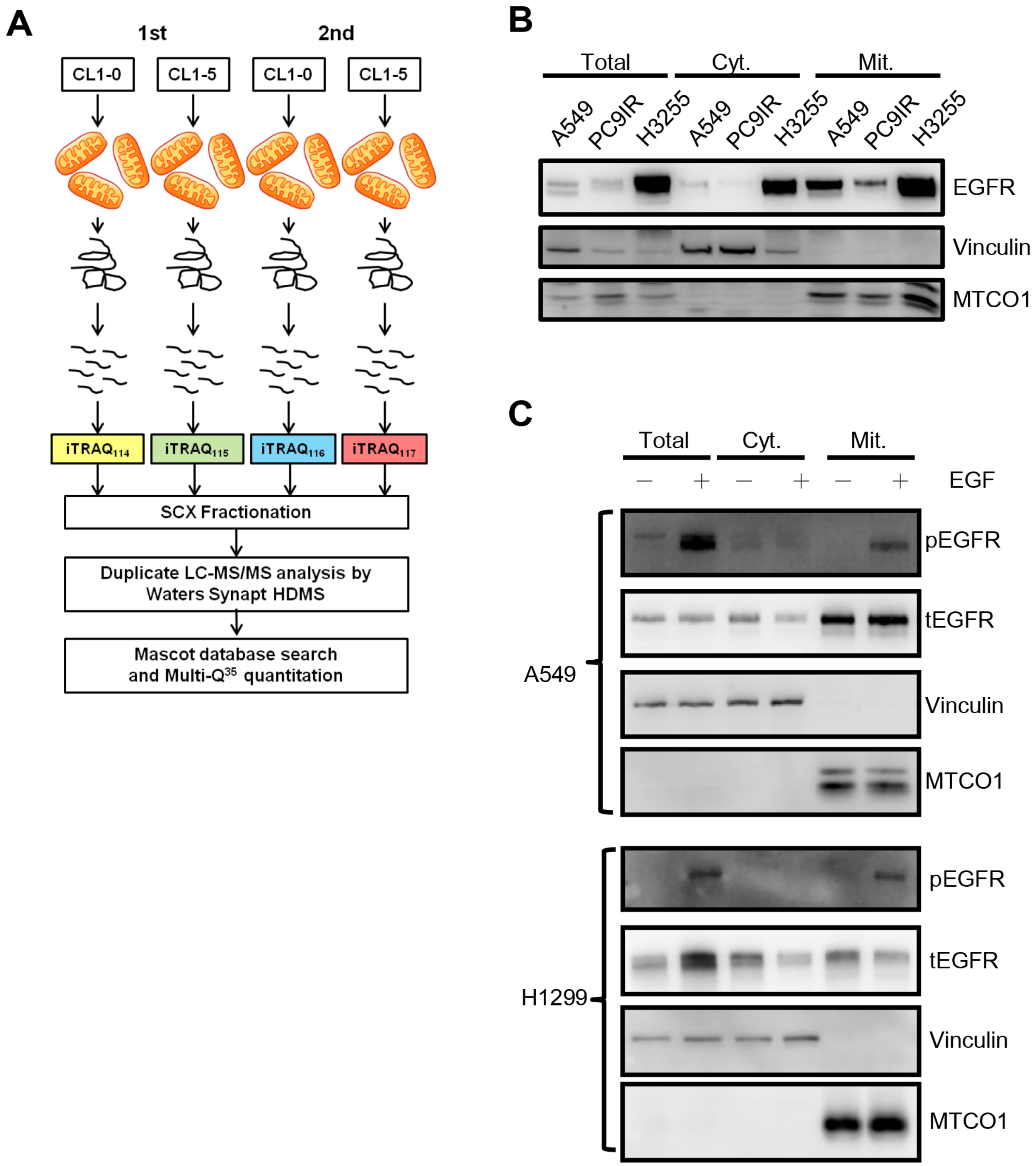

Supplementary Figure S1: A. The flowchart represents the steps of mitochondrial proteomes analysis. Mitochondria of CL1–0 and CL1–5 were purified for iTRAQ analysis, and performed in duplicate. **B.** The mitochondria of A549, PC9IR and H3255 cells were subtracted, and the fractions were subjected to Western blotting. **C.** After serum starvation for 24 h, A549 and H1299 cells were treated with 40 ng/ml of EGF at 37°C for 30 min, and then subjected to the mitochondrial fractionation and immunoblotting. Vinculin and MTCO1 serves as a membranous, cytosolic marker and a mitochondrial marker, respectively.

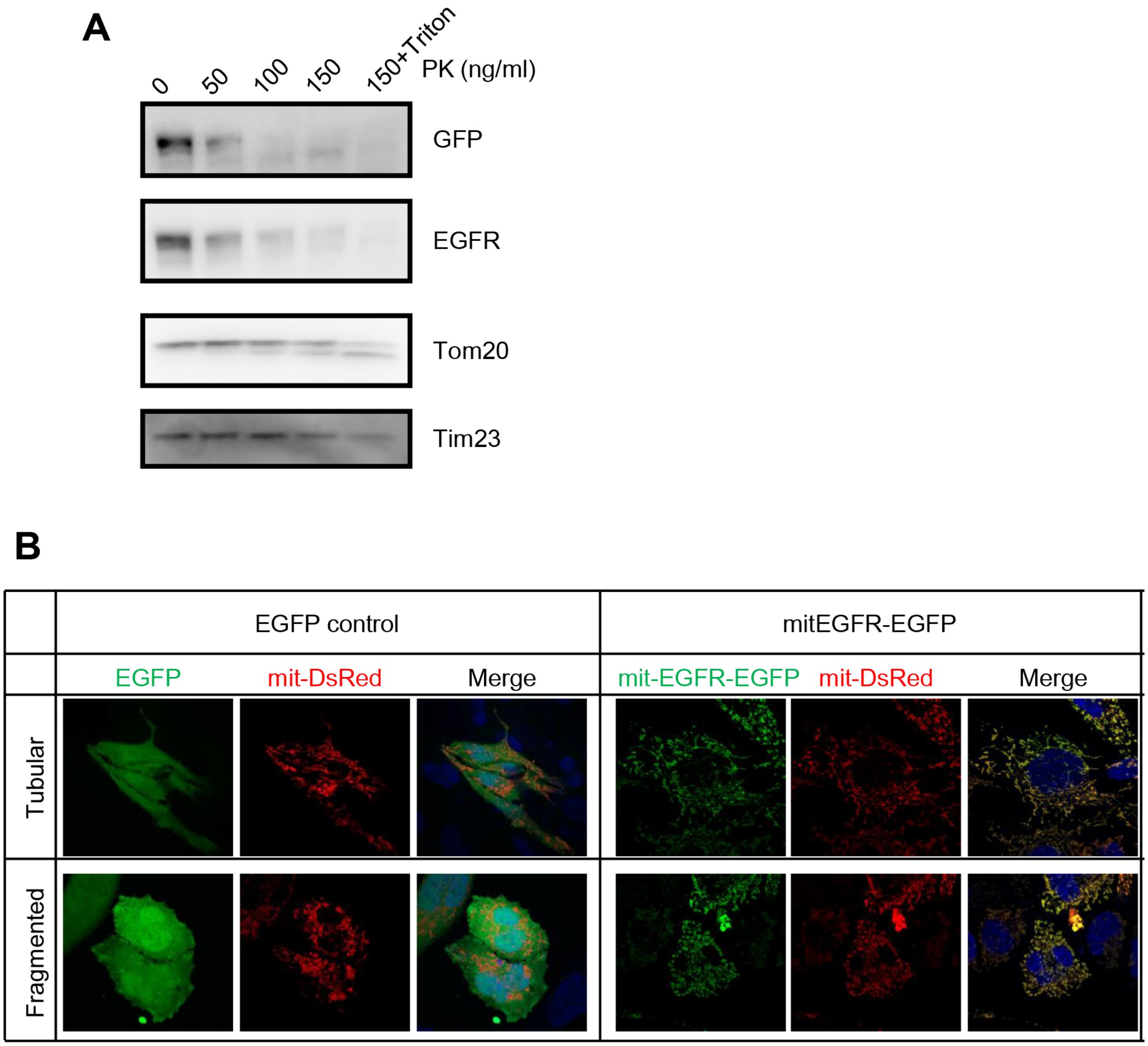

Supplementary Figure S2: A. The localization of the mitochondria of CL1–0 cells transfected with mitEGFR-EGFP plasmids was analyzed by PK digestion. The intact mitochondria were incubated with the indicated concentration of PK at 25°C for 30 min, and then the mixture was subjected to Western blotting. Tom20 and Tim23 serve as the markers for the outer membrane and inner membrane of the mitochondria. **B.** CL1–0 cells were transfected with pMitoDsRed plasmids, and EGFP control vector or mitEGFR-EGFP, and incubate for 24 h. Two groups of the transfected CL1–0 cells were showing different types of the mitochondrial morphology.

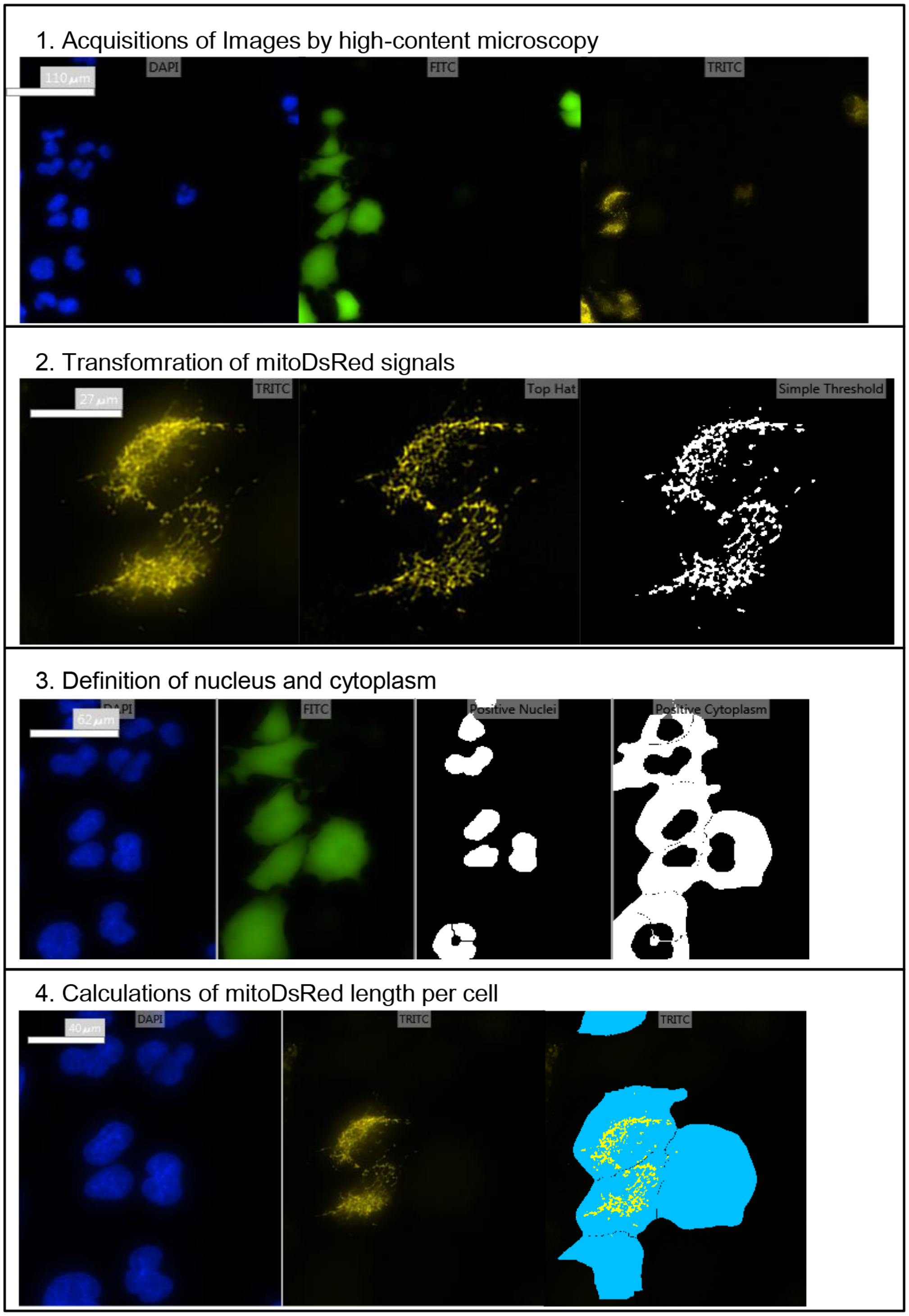

Supplementary Figure S3: The steps of the analysis of mitochondrial length by MetaXpress software

1. The images were acquired by ImageXpress Micro XL System.
2. The signals of mitoDsRed (TRITC) were extracted and transformed into the Top-hat elements analyzed by MetaXpress software.
3. The signals of Hoechst33342 (DAPI) and CFSE (FITC) were defined as a nucleus and the cytoplasm. The addition of nuclear and cytosolic signals was defined as a cell. The cells in the margin of the image were excluded.
4. The mitochondrial length per cell was calculated by MetaXpress software.

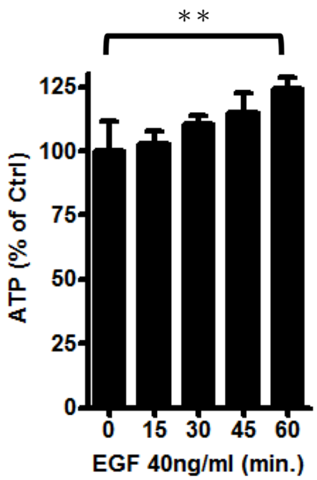

Supplementary Figure S4: After serum starvation for 24 h, CL1–0 cells were then treated with 40 ng/ml of EGF for 0, 15, 30, 45, and 60 min in triplicate. The cellular ATP estimation was performed. Mean ± s.d. is shown, $^{**}P < 0.001$ by Student's *t*-test.

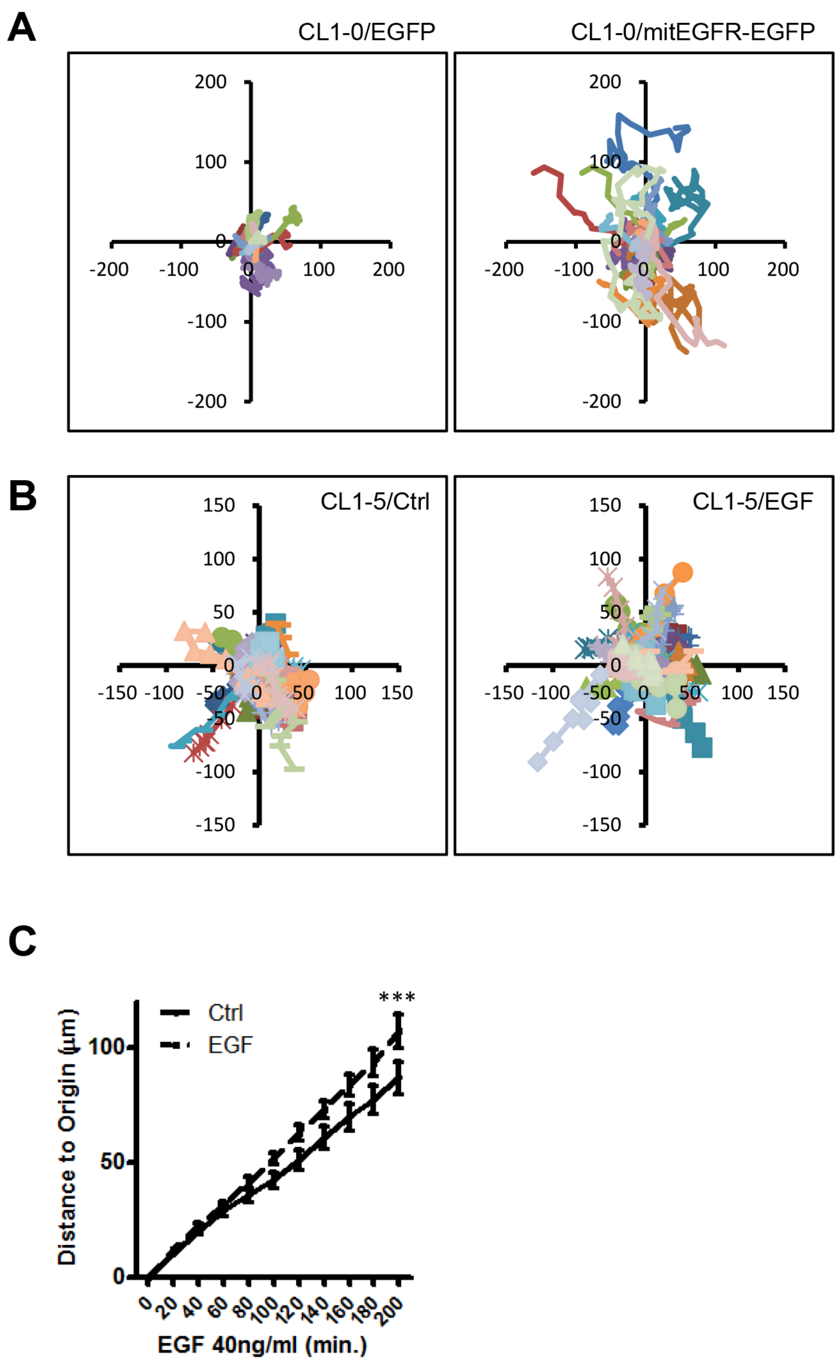

Supplementary Figure S5: A. The tracking routes of the migrated CL1–0 cells were presented in the square, and the left and right panels are represented as EGFP and mitEGFR-EGFP cells, respectively. **B.** and **C.** CL1–5 cells were starved for 24 h. and treated with 40 ng/ml of EGF for recording images every 20 min with the time-lapse fluorescence microcopy. The routes **(B)** and the distance to the origin **(C)** of the migrated cells for 200 min. are shown, and the cell number of untreated group and EGF-treated group is 26 and 38, respectively. Mean ± s.d. is shown, ***P < 0.0001 by Student's *t*-test.

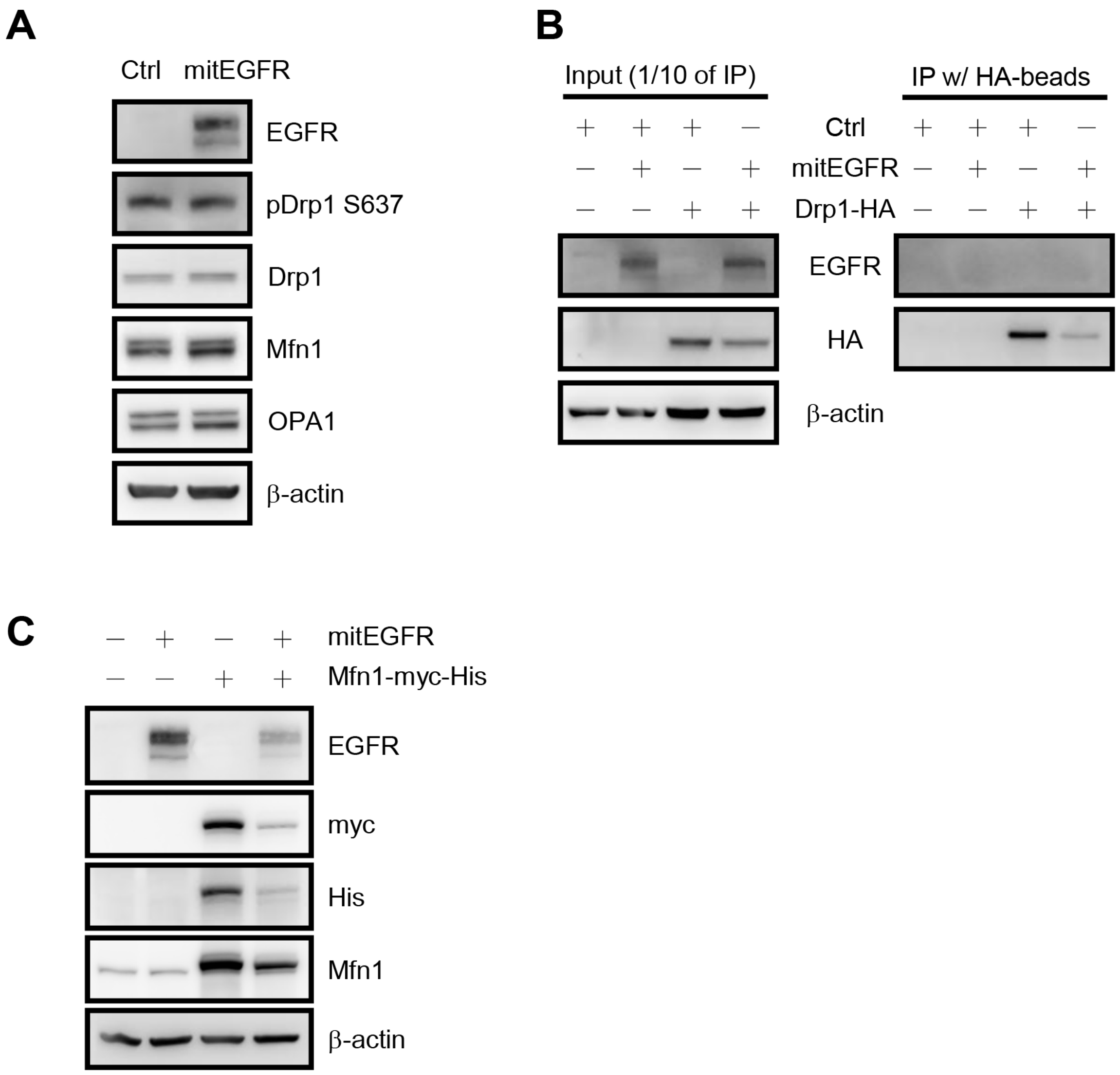

Supplementary Figure S6: A. The lysates of CL1–0 cells transfected with the control and mitEGFR plasmids were subjected to Western blotting for confirming the expression levels of Drp1, Mfn1, OPA1 and phosphor-Drp1. **B.** Co-immunoprecipitaion assay of mitEGFR and Drp1-HA. The lysates of CL1–0 cells co-transduced with control, mitEGFR or Drp1-HA were incubated with anti-HA agarose beads at 4°C for 5 h, and then the mixtures were subjected to Western blotting. **C.** The confirmation of protein expression in CL1–0 cells co-transduced with control, mitEGFR or Mfn1-myc-His plasmids by Western blotting.

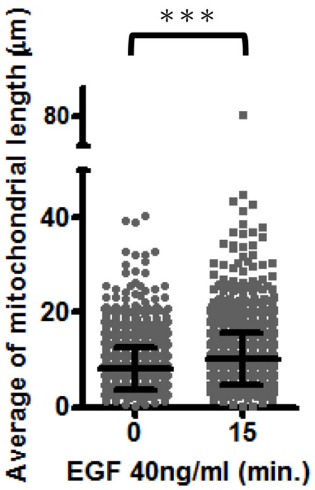

Supplementary Figure S7: The analysis of the mitochondrial length of PC3 cells treated with EGF. PC3 cells were seeded in 96-well, and then starved for 24 h. The cells were treated with 40 ng/ml of EGF at 37°C for 30 min. The mitochondrial length was analyzed by the high-content microscopy. Mean ± s.d. is shown, ****P* < 0.0001 by Student's *t*-test.

Supplementary Table S1: Proteomic analysis of the mitochondria in CL1–0 and CL1–5

1st (CL1–5/1–0)	2nd (CL1–5/1–0)	Gene Symbol
5.08[+-]0	7.37[+-]0.5	RRBP1
5.28[+-]1.45	5.72[+-]1.85	SLC2A1
5.57[+-]1.67	4.1[+-]1.69	MYOF
**6.84[+-]0**	**10.7[+-]3.08**	**EGFR**
8.47[+-]6.01	3.27[+-]1.27	ANPEP
8.76[+-]0.81	7.45[+-]0.69	ASPH

The table showed the examples of the higher protein levels in the mitochondria of CL1–5 cells than in that of CL1–0 cells and the ratio of the protein levels in CL1–5 vs. CL1–0 is presented.

Supplementary Table S2: Clinical characteristics of 29 NSCLC patients evaluated for cytosolic EGFR expression

Characteristics	High cytosolic EGFR expression	Low cytosolic EGFR expression	*P* value
**Age (mean ± SD)**	59.7 ± 12.7	59.4 ± 9.2	0.93[+]
**Gender**	Patients no. (%)	Patients no. (%)	
Male	8 (27.6%)	8 (27.6%)	0.57[§]
Female	7 (24.1%)	6 (20.7%)	
**Stage**			
II	10 (34.5%)	8 (27.6%)	0.44[§]
III	5 (17.2%)	6 (20.7%)	
**Histology**			
Adenocarcinoma	4 (13.8%)	6 (20.7%)	0.24[§]
Squamous cell carcinoma	10 (34.5%)	6 (20.7%)	
Others	2 (6.9%)	1 (3.4%)	

[+]Student's *t*-test
[§]Fisher's exact test

Supplementary Table S3: Multivariate Cox regression analysis of cytosolic EGFR levels and overall survival in 29 NSCLC patients

Variable	Hazard ratio (95% C.I.)	*P* value
Stage	2.04 (2.82–1.47)	0.03
Cytosolic EGFR	1.02 (1.02–1.01)	0.047
